# A community based participatory approach to improving health in a Hispanic population

**DOI:** 10.1186/1748-5908-6-38

**Published:** 2011-04-11

**Authors:** Michael F Dulin, Hazel Tapp, Heather A Smith, Brisa Urquieta de Hernandez, Owen J Furuseth

**Affiliations:** 1 Department of Family Medicine, Carolinas HealthCare System, 2001 Vail Avenue, Charlotte, NC 28207 USA; 2 Department of Geography and Earth Sciences, University of North Carolina at Charlotte, 9201 University City Blvd., Charlotte, NC 28223 USA; 3 Metropolitan Studies and Extended Academic Affairs, University of North Carolina at Charlotte, 9201 University City Blvd., Charlotte, NC 28223 USA

## Abstract

**Background:**

The Charlotte-Mecklenburg region has one of the fastest growing Hispanic communities in the country. This population has experienced disparities in health outcomes and diminished ability to access healthcare services. This city is home to an established practice-based research network (PBRN) that includes community representatives, health services researchers, and primary care providers. The aims of this project are: to use key principles of community-based participatory research (CBPR) within a practice-based research network (PBRN) to identify a single disease or condition that negatively affects the Charlotte Hispanic community; to develop a community-based intervention that positively impacts the chosen condition and improves overall community health; and to disseminate findings to all stakeholders.

**Methods/design:**

This project is designed as CBPR. The CBPR process creates new social networks and connections between participants that can potentially alter patterns of healthcare utilization and other health-related behaviors. The first step is the development of equitable partnerships between community representatives, providers, and researchers. This process is central to the CBPR process and will occur at three levels -- community members trained as researchers and outreach workers, a community advisory board (CAB), and a community forum. Qualitative data on health issues facing the community -- and possible solutions -- will be collected at all three levels through focus groups, key informant interviews and surveys. The CAB will meet monthly to guide the project and oversee data collection, data analysis, participant recruitment, implementation of the community forum, and intervention deployment. The selection of the health condition and framework for the intervention will occur at the level of a community-wide forum. Outcomes of the study will be measured using indicators developed by the participants as well as geospatial modeling.

On completion, this study will: determine the feasibility of the CBPR process to design interventions; demonstrate the feasibility of geographic models to monitor CBPR-derived interventions; and further establish mechanisms for implementation of the CBPR framework within a PBRN.

## Background

The US economy currently depends upon over 35 million immigrant workers who have played a central role in building the country's infrastructure and have filled essential service jobs [1,2]. Despite their contribution, this vulnerable population has, for a variety of reasons (including type of employment and documentation status), been disenfranchised from many essential services including medical care [3]. The majority of US immigrants are Hispanic -- now the largest ethnic minority in the country [4]. Hispanic community members, especially if they are foreign born, are underserved in terms of healthcare and are more likely to be uninsured than any other racial/ethnic group [5]. Although this group bears a disproportionate burden of diseases or conditions such as hypertension, diabetes, and HIV/AIDS, Hispanic immigrants are the least likely to access preventative health services [3,5].

**Figure 1:**
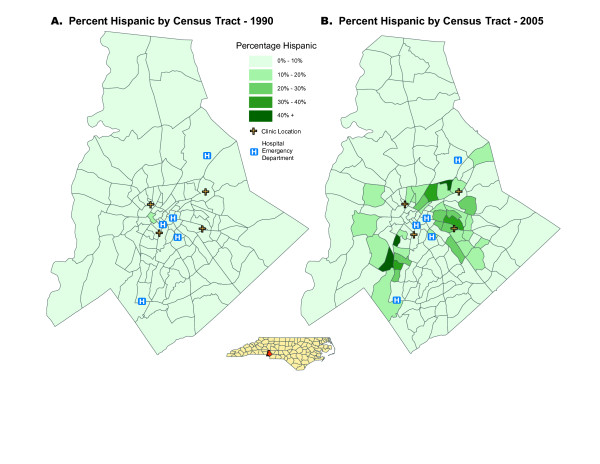
**Maps showing the growth of the Hispanic population in Mecklenburg County between 1990 and 2005**. **Map A **demonstrates minimal Hispanic penetration into the county in 1990. The safety-net clinics are labeled (+) along with the hospital emergency departments (H). **Map B **reveals the striking increase in the Hispanic population by the year 2005. Use of maps such as this will be a key step in engaging participants in the research project.

National data are reflected in Charlotte, North Carolina which, with a 1,404% increase in Hispanic residents between 1990 and 2009 has one of the highest Hispanic growth rates in the nation (Figure 1) [1,6] accompanied by an estimated 65% to 70% Hispanic uninsured rate [4,7]. Many barriers prevent this vulnerable and largely immigrant population from accessing medical care, negatively affecting overall community health [8-10]. Charlotte provides an ideal setting in which to identify new ways to counter barriers and improve health outcomes for Hispanic immigrants. Indeed, as a pre-emerging immigrant gateway, Charlotte has a unique opportunity to create constructive relationships between medical providers and the Hispanic community to proactively and positively impact community health, improve cultural understanding, and break down barriers between community members and health providers.

An essential step to achieve these goals is the use of community-based participatory research (CBPR) within a practice-based research network (PBRN) to build partnerships between researchers, health providers, and community members to inspire social change, restructure service delivery, and improve community health [11-17]. CBPR can employ a wide range of methodologies [14], but key principles include: fostering trusting relationships with community partners; building on strengths and resources within the community; promoting co-learning and capacity building among all partners; utilizing equitable processes and procedures; using cyclic and iterative processes to develop partnerships and build the research process; disseminating results to all partners; involving key stakeholders in all aspects of the research process from the outset; and ongoing partnership assessment, and improvement [13,18-21].

Although CBPR has been offered as a means of promoting community relationships and providing a framework for designing community interventions, there are only a handful of published studies that demonstrate the feasibility of CBPR to influence healthcare outcomes [22-25]. PBRNs are designed to help clinicians better understand and overcome obstacles facing primary care providers as they seek to improve community health. Integrating community participation within a network of providers has been suggested as a way to bridge the gap between the medical system and the community. However, there is a paucity of data available on how to most effectively use CPBR within PBRNs [11].

Although the feasibility of a CBPR approach is often assumed, it is difficult to quantify [18]. Indeed, a review of over 60 CBPR studies was unable to determine the extent to which results that positively affected health outcomes were related (solely or otherwise) to the use of participatory techniques [13].

This paper describes how our team designed a research study using principles of CBPR from the outset with the goal of improving the health of Hispanic immigrants in our community. The goal will be accomplished by the completion of three primary aims: to plan an intervention that positively impacted health outcomes for a specific disease or condition identified by the community; to implement and evaluate the intervention designed in aim one using principles of CBPR; and to disseminate findings to the community and health providers. The study was also designed to: determine the feasibility of the CBPR process to design interventions and evaluation strategies; demonstrate the use of geographic information systems (GIS) models to monitor interventions designed using CBPR; and establish mechanisms for implementation of CBPR principles within a PBRN.

## Methods/design

This study was funded by the National Institutes of Health #R24MD004930 and received ethics approval from the institutional review board of Carolinas HealthCare System #11-09-09E.

### Description of all interventions

**Figure 2:**
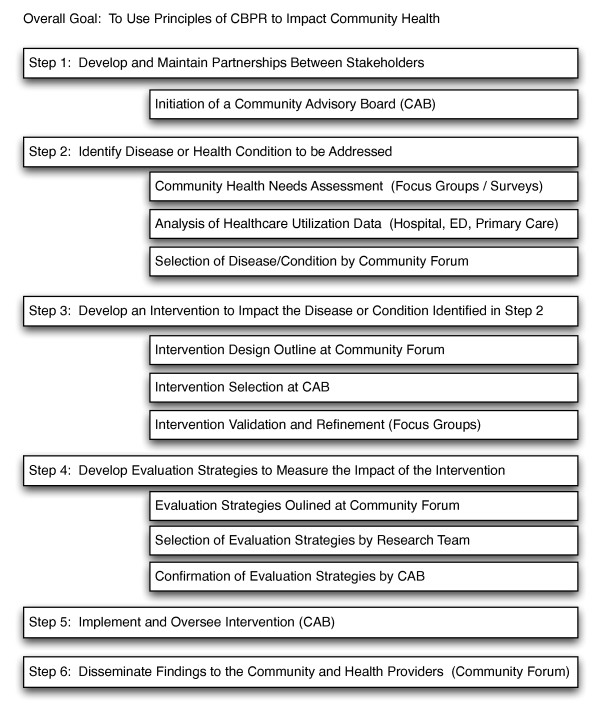
**Study design overview: flow diagram of CBPR guided intervention development**.

This project is designed as CBPR. The CBPR process creates new social networks and connections between participants that can potentially alter patterns of healthcare utilization and other health-related behaviors. In addition, effective utilization of the CBPR process in this project will produce a community-based intervention designed to impact a disease or condition identified by the community as a significant concern. (Figure 2).

### Setting

Community involvement is implemented at multiple levels within this study. The concept for the project was developed and reviewed by a preexisting community advisory board (CAB) within a PBRN (The Mecklenburg Area Partnership for Primary Care Research, MAPPR). This CAB includes representatives from community-based organizations, community members, health providers, and research team members. Collectively, the CAB developed measurement tools using community partnerships that will be of key importance for evaluating this project. These include indicators of community health that can be monitored to determine the feasibility of the developed intervention, and geospatial models that can measure patterns of healthcare utilization for the community (Figures 1 and 2).

The target community is the Hispanic population residing in Mecklenburg County, North Carolina and their healthcare providers. The Hispanic community was chosen because of the tremendous growth of this population, resulting in significant challenges for both the community members and their potential healthcare providers. Hispanic community members in 2010 make up just over 11% of the total Charlotte-Mecklenburg population, or approximately 95,000 people. While there is no reliable data on the census undercount for Hispanics, informal estimates indicate that the census only includes 50% to 60% of the actual immigrant numbers.

Immigrants coming to North Carolina are increasingly migrating directly from rural areas of Central America, with the majority coming from Mexico. Compared to other immigrant groups, those from rural Mexico and Central America have been shown to suffer from greater economic and medical hardships [26], including low rates of medical insurance coverage and low levels of healthcare access [27]. Furthermore, the North Carolina Hispanic population has the lowest rates for routine medical care of any ethnic group in the state (41.1% Latinos without care versus 7.3% for African-Americans and 13.7% for whites) [28]. Charlotte-Mecklenburg Schools (CMS) data further reflect the transition of this county's population, with Hispanic school enrollment growing from 4.5% to 14% of all students between 2000 to 2007 [29]. Even more critical for the future, the greatest number of Hispanic students is found at the elementary school level.

Data from the Mecklenburg County Health Department show that 2007 birth rates were naturally increasing among this population, with one in five of Charlotte area newborns being Hispanic despite their lower representation in the overall population [29]. Economic hardship is another significant factor affecting Charlotte's Hispanic immigrants. Recent data indicate that about 24% of the Hispanic population lives at or below the poverty level and that, on average, Latinos make only about 81.5% of the citywide mean income. During the past four years, medicaid assistance for Latino children grew by 115%, resulting in 16% of all local Medicaid clients being Hispanic in 2008 [11,30].

Data from the local health department and North Carolina Behavioral Risk Factor Surveillance System (BRFSS) 2006 survey shows that the Charlotte/Mecklenburg Hispanic community demonstrates disparities in the following diseases and conditions: immunization rates; access to first trimester prenatal care; HIV infection and HIV-associated death; death from motor vehicle accidents and homicide; teen pregnancy; sexually transmitted infections; overweight children; and percentage of adults who do not participate in physical activity [31].

### Existing involvement with community-based organizations

This project will take place within the MAPPR network and will build upon the existing infrastructure and partnerships. This PBRN was designed from its inception to bring together primary care providers, researchers, and community representatives to study health disparities using key principles of CBPR. The addition of community participation has been identified as an essential next step for PBRN studies [11,32]. However, the mechanisms for successfully implementing CBPR principles within a research network have not been clearly elucidated. Our study, which relies on developing and maintaining strong community partnerships within the PBRN, will provide guidance for other networks as they add the dimension of community participation to their research endeavors.

This research network is based in the Carolinas Medical Center Department of Family Medicine. Member organizations include: primary care clinics, local Hispanic advocacy organizations; churches; The Mecklenburg County Health Department; The UNC-Charlotte Department of Geography and Earth Sciences; The UNC-Charlotte Metropolitan Studies Unit; Mecklenburg County Mental Health; and Charlotte Mecklenburg School Health. The network's community clinics care for over 85% of the city's uninsured patients and had over 194,000 visits in 2008. These clinics, in addition to the county health department and five area hospitals, serve the majority of the city's disadvantaged patients and all are part of a large, vertically integrated healthcare system (Carolinas Healthcare System) that shares a common informatics system. Each participating organization is represented on the CAB that will provide oversight for this research project. Working together, the MAPPR network and member organizations have the potential to significantly improve Hispanic immigrant and overall community health.

## Development of the intervention

### Quantitative data collection

To identify the most common health problems for the Hispanic community, in advance of the start of the project, the research team pulled 2008 data from 307,600 visits to the hospital system's emergency departments (EDs) and primary care clinics. Visits were limited to Hispanic patients living within the targeted community and sorted by diagnosis code (Table 1). In addition, the team will review North Carolina BRFSS results; data collected through focus groups with providers and community members; and answers provided to a community survey. These data will serve as the foundation for the community needs assessment and subsequent identification of the disease/condition that will be addressed by the intervention. Of note, there is significant variation depending upon the data source. The ED data are consistent with our analysis showing that between 60% and 70% of all Hispanic ED visits are for primary care treatable illness. The clinic and ED diagnosis are not necessarily reflective of disparities, but instead show the most commonly occurring visit types.

**Table 1:** Top Five Hispanic Community Health Issues By Collection Site or Methods

	Hispanic Disparities per NC BRFSS	Clinic Diagnoses (n = 5,402)	ED Diagnosis (n = 19,962)	Focus Groups and Interviews (n = 77)	Community Survey (n = 200)
**1**	HIV Infection	Routine Medical Exam	Upper Respiratory Infection	Need for Primary Care Access	Car Accidents

**2**	Death from Motor Vehicle Accidents and Homicide	Upper Respiratory Infection	Abdominal Pain	Prenatal Care	Prenatal Care

**3**	Access to First Trimester Prenatal Care	Viral Infection	Otitis Media	Mental Health / Depression	Mental Health / Depression

**4**	Immunization Rates	Otitis Media	Fever	Substance Abuse	Sexually Transmitted Infections

**5**	Obesity / Overweight	Abdominal Pain	Vomiting	Sexually Transmitted Infections	Assault / Homicide

## Identification of health issues facing the Charlotte Hispanic community

### Community health needs assessment

**Figure 3:**
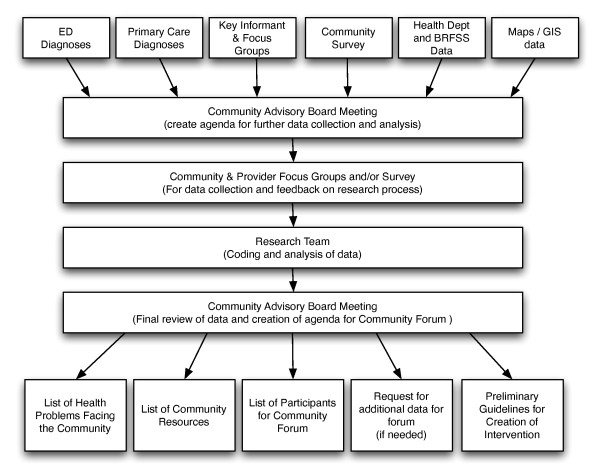
**Flow diagram of data collection and processing plan for community needs assessment**.

The community needs assessment will be directed by the CAB as outlined in Figure 3. This assessment will start with reviewing healthcare data, including the most frequent diagnoses from the ED and primary care clinics for Hispanic patients as well as the results from the baseline key informant interviews, focus groups, and community survey (see Table 1). The CAB will compare these data with the health department data and BRFSS data indicating disparities for the Hispanic community. The CAB and research team will then use these data to develop additional scripts for key informant interviews and focus groups and/or surveys if needed. Data will be coded and analyzed by the research team and made available to the CAB. During this meeting, these data will be used by the board to design the community forum. The product from this meeting will be: a list of health issues facing the community; a list of community resources; a list of potential participants for the community forum; a request for additional data collection; and preliminary guidelines for creation of the intervention.

### Using results from community needs assessment to identify the disease or condition to be addressed by the intervention

A community forum will identify the disease or condition for the intervention. This forum will occur in a community venue and involve approximately 50 participants; real effort will be made to attract a broad representation without prior affiliation to the PBRN. The event will be organized and led by members of the CAB and research team. The preliminary design is based on prior events created by our network, but may be modified by the CAB. The 50 participants will be divided into 10 groups of at least five members each. A member of the CAB will join each group to help clarify any questions about the agenda or the data. The groups will be given three main tasks: to identify a disease or health condition for the intervention; to prioritize guidelines for the intervention; and to recommend two locations in which the intervention might take place. Each group will receive contextual data needed to complete the task. They will be asked to discuss these data as a group and then determine their individual answer to each of the questions/tasks. An audience response system will then be used to anonymously collect the responses to each question/task and immediately provide the tallied results back to the group. This will allow the audience to know what disease/condition has been chosen prior to their responses about prioritizing guidelines and locations. Finally, forum participants will be asked to provide feedback about the meeting on an anonymous paper survey. This will determine their satisfaction with the meeting; ask for feedback to assist the team with development of the second community forum; and seek to determine if participants felt that they had enough information and/or determine what additional data might have been needed for an even more effective meeting.

### Using principles of CBPR to design an intervention that will improve health outcomes for the Charlotte Hispanic community

The community forum will: provide a disease or condition that will be central to the design of the intervention; prioritize guidelines for the intervention; and identify two locations in which the intervention will occur. This information will be reviewed by the CAB, and the research team will start a search to find information about other community-based interventions designed around this disease process. The team will perform a standard literature search and search http://clinicaltrials.gov to see if other groups have started similar projects. The results of this search will be provided to the CAB for review, and a preliminary intervention design will be produced.

Next, focus groups will be used to refine and develop the intervention. The CAB will develop a framework for the composition of the three focus groups (two community, one provider) and their agendas. For example, if the selected condition is depression, and a prioritized guideline is church-based interventions, the CAB/research team could seek participation from community members with depression for the initial community focus group, community church leaders for the second group; and mental health providers for the third group. To continue building and enhancing the rigor of the CBPR process, representatives from each of these focus groups will be invited to join the CAB for the remainder of the project. Transcriptions and summaries of the feedback from the focus groups will then be provided back to the CAB for review, and based on this information the CAB will finalize the intervention design.

## Analysis

### GIS analysis of the patterns of healthcare access

**Figure 4:**
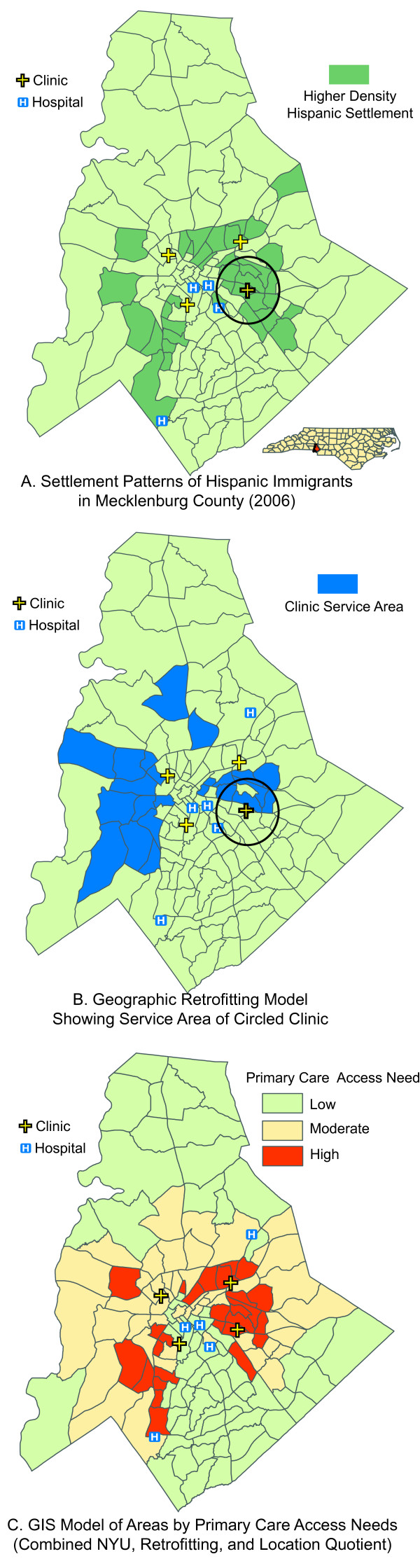
**Sample geospatial models showing patterns of community healthcare utilizations**. **Map A**. Hispanic settlement patterns by census tract (target clinic noted with circle). **Map B **The geographic retrofitting model demonstrates the actual service area for the target clinic (note - many patients come to the clinic from distant parts of the city). **Map C **Complete models showing areas in need of improved access to primary care based on the retrofitting model of the safety-net, settlement patterns, and inappropriate ED utilization identified by the NYU algorithm.

This project will use GIS and geographic retrofitting as a means to evaluate the intervention's impact over time. GIS has the power to map variables within a community to demonstrate spatial relationships between health predictors and outcomes [33-35]. While mapping tools have long been used to track health-related factors such as disease transmission, less common has been their use to effectively evaluate patterns of healthcare access and to define community service areas [36-38]. However, these tools can also be used effectively to evaluate patterns of healthcare access and to define community service areas [39]. GIS models of provider penetration into a community are robust enough to withstand quantitative analysis and to define inequalities in delivery of medical services [40]. The research team has successfully used a combination of GIS tools to create models showing past, current, and projected patterns of healthcare access at the community level (Figure 4)[41,42].

Geographic retrofitting defines the service areas of medical facilities allowing for analysis of service delivery and intervention design [40]. This model works by dividing the number of clinic patients in a given census tract by the total population in the tract. A histogram of the resulting information is created by adding each census tract into the defined community until a 50% threshold is reached starting with areas of highest use.

The New York University (NYU) ED Algorithm was developed by Billings and colleagues (2000) as an indicator of the ability of a local safety-net to provide primary care services [43-45]. Following this model, all ED data for our project will be geo-coded every six months, and the NYU algorithm will be used to sort the data, and results will be mapped using ArcGIS (ESRI, Redlands, CA). Maps will be divided by race/ethnicity to find areas where Hispanic residents over utilize emergency services for primary care treatable illness.

The research team will use GIS tools to create models showing patterns of healthcare access across the community. An example is shown in Figure 4, where we examined clinic locations and compared them with Hispanic settlement patterns. For example, 20% of the city's Hispanic population lives within a three-mile radius of the clinic circled in Figure 4A, but only 4% of the clinic's patients were Hispanic. Second, a geographic retrofitting model was applied to clinic data to identify clinic service areas. This is seen in Figure 4B, where patients at the sample clinic traveled an average of over 9.5 miles to receive care. All community clinics underwent a similar analysis that, once combined, provided a comprehensive map of the community's medical safety-net. Third, the NYU algorithm, an estimate of inappropriate ED utilization for primary care treatable conditions, was used in combination with the safety-net map to create a model of primary care needs for the county (Figure 4C).

This model of community primary care need is sensitive to community-wide changes in both primary care and ED utilization. This model will be recreated at baseline and every six months for the duration of the project to assess potential changes in access that may be occurring as a result of the intervention.

### Development of additional evaluation strategies to measure the impact of the intervention

After the disease and intervention are chosen, impact measurement strategies will be developed. The CAB will review and approve the final design of the intervention, and subsequently work with the research team to identify evaluation strategies to define the success of the intervention. They will be able to draw on the network's ability to access extensive clinical data from the hospital, ED, primary care clinics, and health department for this evaluation. If possible, these data will also be geo-coded and mapped as part of the analysis. Examples could include: number of Hispanic patients diagnosed with sexually transmitted infections in the intervention ED versus the control ED; blood pressure measurements for Hispanic patients in the intervention primary care clinic versus control; or number of patients from one geographic region with a diagnosis of depression identified at the health department before and after the intervention.

### Implementation and evaluation of the intervention using principles of CPBR to implement the intervention with community supervision and feedback

The CAB and research team along with additional invited community representatives will direct the intervention throughout the remainder of the project. This collaborative group will meet monthly and rely upon member input and resources to implement and monitor the intervention.

To determine the impact of the intervention, preliminary research identified prenatal care, mental health, substance abuse, and sexually transmitted infections as community health concerns. The CBPR process will allow us to confirm and augment this list. In addition to the disease or condition selected through the process, selected variables from this list will be followed through the course of this project as a way to track community health. All outcome variables will be followed at least every six months (or more often, if desired by the CAB).

Initial studies by the research team have used geospatial models of primary care and ED services to monitor changes in primary care access during the CBPR planning process. These models will be used to prospectively monitor community-wide changes in primary care access as a result of the CBPR process used in this proposal. Changes that enhance primary care utilization have the potential to broadly impact community health, making this an essential step in the evaluation process [46,47].

Qualitative feedback about the intervention will be obtained from four additional focus groups that occur during different stages of the intervention. These groups will consist of community representatives and health providers in both the control and intervention groups. The focus group agendas will be designed by the CAB and research team, and will be focused on collecting data that can assess the intervention's impact and sustainability. Focus group data will also be used as necessary to make adjustments to the intervention as it is implemented.

### Dissemination of findings to community and provider partners

In addition to the sustainability of the intervention(s), we seek to ensure the sustainability of the community and provider partnerships that are at the core of successful efforts to reduce health disparities. As such, findings from this study will be shared with these partners and their broader communities in a number of ways. First, a final community forum will be held at the end of the pilot intervention. Again, the community forum composition and agenda will be designed by the CAB in consultation with the research team. The main purpose of this event will be to solicit feedback about the intervention and disseminate findings from the project to all community and provider partners. The team will use the audience response system to anonymously collect and present tallied responses to structured feedback questions about the intervention and project findings. The last agenda item at this forum will bring researchers, providers, and community partners together to talk about prioritizing and structuring manuscripts for peer review to both social science and medical outlets, as well as generating ideas and task lists for follow-on research projects and applications to future funding agencies. Second, an executive summary of the project, its outcomes, and recommendations (which will include feedback received from the forum) will be prepared by the research team and distributed to each partner in paper and electronic format. Versions will also be posted on the MAPPR and UNC-Charlotte Metropolitan Studies websites. Further, this executive summary will form the basis for a series of presentations that will be prepared and delivered to community and provider groups as well as to broader public constituencies as requested.

## Discussion

This paper describes a protocol using the participatory approach that will be used to advance community health through the development of a research protocol that aligns with the healthcare needs of the targeted community. Although the process outlined here engages and partners with the community to identify the disease and build the intervention from the ground-up, there are still some limitations.

First, when working with a transitioning immigrant community, there is a likelihood of participants leaving both the project and the city, necessitating the recruitment of new participants as the project unfolds. This is mitigated by the protocol design that provides three levels of community participation (the CAB, community forums, and collection of data via survey and focus groups). However, turnover at the level of the CAB in particular can be a challenging issue.

Second, research team members tend to be more outspoken and willing to take leadership positions within the CAB. Our team continues to work to identify ways of increasing the levels of equitable partnership and contribution at the CAB level. Indeed, we are increasingly cognizant that this level of CBPR requires continuous process assessment and improvement to be both effective and sustainable.

Despite these limitations, facilitating community involvement throughout a CBPR process has many benefits including but not limited to: facilitation of recruitment, enriched data collection, more rapid analysis, and translation of results from the study back into the community. In particular, we feel that the intervention developed through this process is more likely to be implemented because of high levels of sustained community engagement and human capital investment in the process. Our team also feels strongly that using participatory methods strengthens and enriches the research process while enhancing the skills and capacity of all participants.

## Competing interests

The authors declare that they have no competing interests.

## Authors' contributions

All authors made significant contributions to the conception and design of this study and read and approved the final manuscript. MD, HT, and HS drafted the manuscript.
